# Genomic and transcriptomic analysis of *Ligilactobacillus salivarius* IBB3154—in search of new promoters for vaccine construction

**DOI:** 10.1128/spectrum.02844-23

**Published:** 2023-11-20

**Authors:** Patrycja Kobierecka, Agnieszka Wyszyńska, Tamara Aleksandrzak-Piekarczyk, Agnieszka Sałańska, Jan Gawor, Jacek Bardowski, Katarzyna Elżbieta Jagusztyn Krynicka

**Affiliations:** 1 Department of Bacterial Genetics, Institute of Microbiology, Faculty of Biology, University of Warsaw, Warsaw, Poland; 2 Institute of Biochemistry and Biophysics, Polish Academy of Sciences, Warsaw, Poland; 3 DNA Sequencing and Synthesis Facility, Institute of Biochemistry and Biophysics, Polish Academy of Sciences, Warsaw, Poland; Forschungszentrum Juelich GmbH, Juelich, Germany

**Keywords:** *Ligilactobacillus salivarius*, promoters, serine-rich repeat protein, bile acid, transcriptomic

## Abstract

Transcriptomic analysis of the genome sequenced *Ligilactobacillus salivarius* strain IBB3154 grown at two different temperatures (37°C vs 42°C) identified differentially expressed genes involved in metabolic pathways, osmoregulation, and surface protein expression. Two highly expressed genes, *sasA1* and *sasA2*, which encode cell wall-anchored proteins belonging to the serine-rich repeat protein group, were found to be temperature-inducible. Moonlighting proteins with various functions, such as glyceraldehyde 3-phosphate dehydrogenase, fructose-bisphosphate aldolase, elongation factor Tu, and enolase, were highly expressed at both temperatures. The efficiency of promoters has been confirmed by the β-glucuronidase activity test; however, temperature dependence was not detected. We also found that the P*_sasA1_* promoter retained its activity in the presence of bile salts. Knowledge of promoters that are highly active in *L. salivarius* cells can be used to produce strains that are carriers of immunogenic proteins.

**IMPORTANCE:**

The genome of the strain *Ligilactobacillus salivarius* IBB3154 was sequenced, and transcriptome analysis was carried out at two different temperatures, allowing the determination of gene expression levels in response to environmental changes (temperature). Genes with higher expression at 42°C were identified. The use of a reporter gene (β- glucuronidase) did not confirm the transcriptomic results; it was found that the promoters of the genes *sasA1* and *sasA2* were active in the presence of bile salts. This opens up new opportunities for the overexpression of genes of other bacterial species in *Ligilactobacillus* cells in the intestinal environment.

## INTRODUCTION

Lactic acid bacteria (LAB) are gram-positive, low-G + C bacteria defined by their common ability to produce lactic acid as the main metabolic product of carbohydrate fermentation. Intensive genetic and molecular research carried out on LAB, mainly *Lactococcus lactis* and some species of the *Lactobacillus* genus (before reclassification), has revealed that these strains can be used as immunostimulants or bacterial carriers of compounds with therapeutic or prophylactic effects (1 – 4). As vaccine vectors, they allow immunization through the mucosal route, which increases effectiveness against pathogens that use the mucosa as the main route of entry into the human or animal body. Administration of LAB strains leads to the induction of an immune response in both mucous membranes and a systemic immune response against expressed heterologous antigens, with a low response against carrier strain antigens (5). The attractiveness of lactic acid bacteria in immunoprophylaxis or therapy is also determined by their resistance to the low pH of gastric juice and their ability to adhere to intestinal epithelial cells (6 – 8). Additionally, the safety of these preparations is a key issue. Many LAB (including species: *Lactobacillus*, *Lactiplantibacillus*, *Ligilactobacillus*, *Lactococcus*, and *Leuconostoc*) have been granted Qualified Safety Presumption status by the European Food Safety Authority and GRAS status (generally recognized as safe) by the U.S. Food and Drug Administration. This recognition is attributed to their extensive history of safe use in fermented foods and their presence in the normal intestinal and urogenital microbiota of both humans and animals (9, 10). Furthermore, some LAB species are acknowledged as probiotics, organisms whose short-term presence or colonization positively influence many elements of the host’s physiology. The undoubted advantage is also the low production costs of this type of vaccine and the possibility of freeze-drying and storage of the preparation at room temperature (5, 11).

Since the first report on the use of lactic acid bacteria as a carrier for vaccine antigens, many studies have been published (12). Attempts have been made to use recombinant LAB strains in immunoprophylaxis against gastrointestinal pathogens (*Salmonella*, *Helicobacter*, *Yersinia*), bacteria that cause respiratory infections (*Streptococcus pneumoniae*) (5), as well as against numerous viruses such as SARS-CoV-2 (13), Human Papilloma Virus (14), rotavirus (15), Porcine Epidemic Diarrhea Virus (16, 17), and subgroup J Avian Leukosis Virus (18). Various strategies have been developed to efficiently express genes encoding heterologous antigens in LAB strains, mainly of the genera *Lactococcus* and *Lactobacillus*. The effectiveness of the preparations varied and was dependent on many parameters, such as the type of antigen (bacterial, parasitic, or viral), carrier strain, amount of antigen, its location, route of administration, and the immunization schedule (17, 19 – 22).

Our research group has been working to construct an efficient chicken anti-*Campylobacter* vaccine, using LAB strains as delivery vectors for *Campylobacter* antigens. Our previous work documented the possibility of using the *L. lactis* strain as a delivery vector for the *C. jejuni* antigen (23). However, since *L. lactis* does not colonize the chicken intestinal tract, we decided to use as a carrier the *Ligilactobacillus salivarius* strain isolated from chicken feces (24). As it is well known that the amount of antigen has an impact on vaccine efficacy, this study was intended to identify new promising candidates for promoter controlling the expression of heterologous proteins in *L. salivarius*. During colonization processes, lactobacilli often face stressful conditions such as temperature changes, acidity, osmolarity, and oxidative conditions. It should be noted that strains of lactic bacteria colonizing chickens can encounter a wide temperature range (chicken body temperature: 42°C); therefore, it is crucial that the promoter strength is not decreased by this factor. Comparison of the transcriptomes of *L. salivarius* IBB3154 cells grown at two different temperatures (37°C and 42°C) allowed the identification of 11 genes whose expression was higher at 42°C and five genes whose expression was very high at both temperatures.

## MATERIALS AND METHODS

### Bacterial strains, primers, plasmids, media, and growth conditions

Bacterial strains, plasmids, and primers used in this study are listed in Supplementary Material (Tables S1 and S2). The *Ligilactobacillus salivarius* IBB3154 strain was routinely cultured in de Man, Rogosa, and Sharpe (MRS) broth or MRS agar (solidified with 1.5% agar) medium (Oxoid) at 37°C without shaking under microaerobic conditions provided by Anoxomat Mark II OP (MART Microbiology B.V.). The *Escherichia coli* strains, used as hosts for the construction of recombinant plasmids, were grown under standard conditions unless otherwise indicated. The *E. coli* strain MC1061 (*recA* ^+^) was used for cloning purposes in pNZ8008. When needed, the medium was supplemented with antibiotics at the following final concentrations: for *E. coli,* 20 µg/mL chloramphenicol, 100 µg/mL ampicillin, and for *L. salivarius,* 10 µg/mL chloramphenicol.

### Bacterial genome sequencing

Whole genome sequencing of the *L. salivarius* IBB3154 strain was carried out in the DNA Sequencing and Synthesis Facility in the Institute of Biochemistry and Biophysics of the Polish Academy of Sciences. Total genomic DNA was isolated using the cetyltrimethylammonium bromide/lysozyme method (25) with minor modifications. The bacterial cell wall was digested enzymatically before lysis with sodium dodecyl sulfate using lysozyme (20 mg/mL; Sigma-Aldrich, Dorset, UK) and mutanolysin (5 U/mL; A&A Biotechnology, Gdańsk, Poland) for 15 min at 37°C. After isolation, DNA integrity was checked by electrophoresis on a standard 1% agarose gel and by pulsed-field gel electrophoresis on a CHEF-DR III apparatus (Biorad Laboratories, Hercules, CA). Genomic DNA concentration was measured by fluorimetry using a Qubit 2.0 (Thermo Fisher Scientific, Waltham, USA). The DNA sample was sheared into approximately 20 kb fragments using a Covaris gTUBE (Covaris Ltd., Brighton, UK), and the sequencing library was constructed using a SMRTbell template prep kit version 1.0. The library sample was loaded into a single single-molecule real-time cell and sequenced on the RS II instrument (Pacific Biosciences, Menlo Park, CA). Additionally, the bacterial genome was sequenced using Illumina technology. Genomic DNA was mechanically sheared to an appropriate size by nebulization and used for Paired-End TruSeq-like library construction using the KAPA Library preparation kit (KAPA/Roche, Basel, Switzerland) following the manufacturer’s instructions. The bacterial genome was sequenced in paired-end mode (v3, 600 cycle chemistry kit) using the MiSeq instrument (Illumina, San Diego, CA). Illumina sequence reads were filtered for quality using the FastX toolkit (http://hannonlab.cshl.edu/fastx_toolkit/). PacBio reads were assembled into contigs using Canu v1.6 (26). The Canu assembly was further polished using Illumina reads and Pilon v1.22 (27). The remaining sequence gaps and ambiguities in genome assembly were closed and verified by the PCR amplification of DNA fragments, followed by Sanger sequencing with an ABI3730xl Genetic Analyzer (Life Technologies, Thermo Fisher Scientific, Waltham, USA), using BigDye Terminator Mix v. 3.1 Chemistry (Thermo Fisher Scientific, Waltham, USA). The genome was manually closed using Seqman software (DNAStar, USA) to obtain a complete nucleotide sequence of the bacterial genome.

### RNA extraction and RNA-seq

RNA extraction and RNA-seq were performed in the DNA Sequencing and Synthesis Facility in the Institute of Biochemistry and Biophysics of the Polish Academy of Sciences. Bacterial cells were inoculated in triplicate in 100 mL of MRS broth. Each triplicate was incubated at 37°C and 42°C for 5 h (OD_600_ ~ 1). Cells were harvested as rapidly as possible by centrifugation at 5,000 rpm for 10 min. The growth medium was removed, and the cells were resuspended in StayRNA reagent (A&A Biotechnology, Gdańsk, Poland), aliquoted into 2 mL portions, and incubated overnight in the freezer. After overnight incubation, the StayRNA reagent was removed by centrifugation, and the bacterial cell pellets were stored at −70°C. After thawing, the bacterial cells were resuspended in 50 µL of Tris-EDTA buffer containing 10 mg/mL of lysozyme (Sigma, USA) and 1 U/mL of mutanolysin (A&A Biotechnology, Gdańsk, Poland) and incubated for 15 min at 37°C. The cells were then centrifuged, and the enzyme cocktail was removed prior to lysis with Fenozol reagent (A&A Biotechnology, Gdańsk, Poland). Total RNA was extracted using the Total RNA Mini Plus kit (A&A Biotechnology, Gdańsk, Poland). The RNA was treated with Rnase-free Dnase (A&A Biotechnology, Gdańsk, Poland) to remove any contaminating genomic DNA, according to the manufacturer’s protocols. RNA quality was assessed using the Agilent 2100 Bioanalyzer system and the RNA 6000 Nano Chip (Agilent Technologies, Santa Clara, USA). The quantity of RNA was measured by fluorimtery using a Qubit2.0 fluorimeter (Thermo Fisher Scientific, Waltham, USA). Ribosomal RNA species were depleted using the RiboZero rRNA (Bacteria) Removal Kit (Illumina, San Diego, USA). The quality of the RNA and the absence of the 16S and 23S rRNA species were quality checked on an Agilent Bioanalyzer 2100 using the RNA 6000 Pico kit.

Six independent RNA-seq libraries were constructed with the KAPA Stranded RNA-Seq kit (KAPA-Roche, Basel, Switzerland) following the manufacturer’s protocol. The quality and quantity of purified libraries were checked using the Agilent 2100 Bionalyzer, Qubit2.0 (Life Technologies, Thermo Fisher Scientific, Waltham, USA), and the KAPA Library qPCR Quantification Kit (KAPA-Roche, Basel, Switzerland) and subsequently sequenced on an Illumina NextSeq 500 instrument with 75 nucleotide single-end reads. The raw reads were trimmed for quality using the FastX toolkit (http://hannonlab.cshl.edu/fastx_toolkit/) and the remaining Illumina adapters were removed using cutadapt (https://github.com/marcelm/cutadapt). Cleaned sequencing reads were mapped to the *L. salivarius* 3,154 genome with CLC Genomics Workbench 9.0.1 (https://www.qiagenbioinformatics.com/), and the rest of the analysis was done with this software package. The statistics of the RNA-seq data set have been presented in the Supplementary Material (Table S3). The abundance of gene expression was normalized by fragments per kilobase of exon per million mapped fragments. Differentially expressed genes (DEGs) were identified, with the threshold set to 1.8-fold change and a *P*-value <0.05 as the criteria for a significant difference in gene expression.

### General DNA manipulations

Standard DNA manipulations were performed as described in the Sambrook manual (28) or according to the manufacturer’s instructions (A&A Biotechnology, Poland). The chromosomal DNA of *L. salivarius* IBB3154 used for PCR reactions was isolated using a commercial kit and protocol (A&A Biotechnology, Poland). Cells were pretreated with mutanolysin for 1 h. PCRs were performed with HotStar HiFidelity Polymerase (Qiagen) or PrimeStar HS DNA Polymerase (Takara) under standard conditions. Synthetic oligonucleotide synthesis and DNA sequencing for cloning experiments were performed by Genomed S.A. (Warsaw, Poland). The DNA fragments containing *Ligilactobacillus* promoters were synthesized by GeneCust (France).

### Construction of recombinant plasmids

The DNA fragments containing the promoter of the *sas1* gene (P*_sas1_*) and the promoter of the *sas2* gene (P*_sas2_*) were synthesized by GeneCust and cloned into pBluescript II SK + digested with PstI and XhoI, generating the plasmids pUWM1476 and pUWM1477, respectively. Then pUWM1477 was digested with BglII and EcoRI, and a 0.5-kb DNA fragment was inserted into pNZ8008/BglII and EcoRI, resulting in pUWM1481. To introduce new sites recognized by restriction enzymes, the DNA fragment containing (P*_sas1_*) was amplified from pUWM1476 with the pair of primers pUWM1476_BglII and pUWM1476_PstI and cloned into pJET 1.2 (Thermofisher, USA). Subsequently, the resulting plasmid, pUWM1486, was digested with BglII and PstI, and a 0.5-kb DNA fragment was inserted into pNZ8008/BglII and PstI, generating the plasmid pUWM1491.

The 0.28-kb DNA fragment that contains the promoter of the *usp45* gene was amplified from the *L. lactis* subsp. *Lactis* IL1403 chromosome (GenBank accession number AE005176) [PrimeStar HS DNA Polymerase (TaKaRa)] using the primer pairs Usp45_BglII and Usp45_PstI. The PCR product was purified and cloned into pJet1.2/blunt to generate pUWM1553. Subsequently, the BglII-PstI DNA fragments of pUWM1553 and pNZ8008 digested with the same enzymes were ligated. The resulting plasmid, designated pUWM1565, contains fragment DNA that includes the promoter of the *usp45* gene.

The plasmid containing fragment DNA with the promoter of the fructose-bisphosphate aldolase (P*_fbaA_*) gene was created in a similar way. Briefly, the 0.5-kb DNA fragment containing the promoter of the *fbaA* gene was amplified from the chromosome of *L. salivarius* IBB3154 [HS DNA Polymerase (TaKaRa)] using the primer pairs Faldo_BglII and Faldo_PstI. The PCR product was purified and cloned into pJet1.2/blunt to generate pUWM1488. Subsequently, the BglII-PstI DNA fragments of pUWM1488 and pNZ8008 digested with the same enzymes were ligated. The resulting plasmid was designated pUWM1498.

### Transformation of *E. coli* and *L. salivarius*

*E. coli* was transformed as previously described (29). Recombinant plasmids pUWM1481, pUWM1491, pUWM1498, and pUWM1565 were introduced into *L. salivarius* cells by electroporation (30). To prepare the competent *Ligilactobacillus* strain cells, an overnight culture was inoculated 1:50 in MRS containing 1% glycine and incubated at 37°C under microaerobic conditions until an OD_600_ of 0.5 was reached. Bacteria were collected (4,000 rpm, 10 min, 4°C), and the pellet was washed three times in electroporation buffer (1 M sucrose, 3.5 mM MgCl_2_). The bacteria were resuspended 1:100 in the same solution, and a volume of 20 µL was immediately electroporated or kept at −70°C for further use. *L. salivarius* competent cells were electroporated at 2.5 kV, 400 Ω, and 25 µF in 0.2 cm cuvettes using a BioRad GenePulser (BioRad, Life Science Research Products, CA, USA). After electroporation, *Ligilactobacillus* cells were resuspended in MRS broth containing 0.5 M sucrose and 100 mM MgCl_2_ and incubated at 37°C for 3 h. After incubation, bacteria were plated on MRS supplemented with chloramphenicol (10 µg/mL). The plates were incubated at 37°C for 2 days.

### β-Glucuronidase activity assay

For the β-glucuronidase (GUS) activity assay, *L. salivarius* IBB3154 cells carrying plasmids encoding β-glucuronidase under the control of tested promoters were used. *Ligilactobacillus* strains were grown in MRS broth supplemented with chloramphenicol and bile salts (0%, 0.05%, 0.1%, and 0.2%) under microaerobic conditions at 37°C or 42°C (25 mL). After reaching log phase growth (A_600_ = 0.6), cells were harvested by centrifugation (4°C, 5 min, 8,600 × *g*) and washed twice in ice-cold GUS buffer (4 mL; 100 mM Na_3_PO_4_ and 2.5 mM EDTA, pH 6.0). Subsequently, the cell pellet was thoroughly suspended in 1 mL of GUS buffer and disrupted by beating the beads for three 1 min cycles, with intervals of at least 1 min, during which the cells were kept on ice (FastPrep Instrument; MP Biomedicals, Santa Ana, CA). After centrifugation (8,600 × *g*, 2 min, 4°C), cell-free extracts were stored on ice and used immediately. Protein concentrations were determined using the bicinchoninic acid protein assay kit (BCA kit; Sigma-Aldrich).

For the determination of β-glucuronidase activity, 100 µL of each cell extract (0.1 mg/mL) were transferred to a black 96-well plate and incubated for 1 min at 37°C. Then, 100 µL of 2 mM MUG (4-methylumbelliferyl-beta-D-glucuronide), a fluorogenic substrate of β-glucuronidase (Sigma-Aldrich), was added, and the mixture was incubated for 10 min at 37°C. After incubation at 37°C for 10 min, 50 µL of 3.2 M Na_2_CO_3_ was added to stop the reaction. Subsequently, fluorescence intensity was measured using a 96-well plate reader at 355 nm excitation and 460 nm emission (Synergy HTX, BioTek). For each experiment, standards were prepared for the GUS assay. To obtain a standard curve (0–250 nM 4-MU), 1 mM 4-MU (4-methylumbelliferyl) stock was diluted to 5, 10, 20, 50, 100, and 200 nM in GUS/Stop buffer (buffer GUS:3.2 M Na_2_CO_3_, 4:1, vol:vol).

Units of GUS activity were described as nanomoles of 4-methylumbelliferone released per minute per milligram of protein. Three biological replicates were performed for all experiments.

### Bioinformatic analyses

Open reading frames (*orf*s) and noncoding RNAs were annotated using the RAST server [http://rast.nmpdr.org/ (31)] and, when needed, checked by BLAST analysis. BASYs [https://www.basys.ca/ (32)] software was used to construct Clusters of Orthologous Groups (COGs) of predicted proteins. Possible bacteriophages were identified using PHAST [http://phast.wishartlab.com/ (33)]. The Clustered Regularly Interspaced Short Palindromic Repeat (CRISPR) loci were searched using the CRISPRFinder tool [http://crispr.i2bc.paris-saclay.fr/ (34)]. Genome visualization was performed using the CGView server [http://cgview.ca/ (35)]. Antibiotic resistance genes were searched using the Comprehensive Antibiotic Resistance Database (CARD) Resistance Gene Identifier (RGI) (36).

### Statistical analysis

Differences in β-glucuronidase activities in tested strains were analyzed with GraphPad Prism 7.0 software (GraphPad Software, CA, USA) using one-way analysis of variance with Bonferroni correction. *P*-values below 0.05 were considered significant.

## RESULTS

### Characteristics of the *L. salivarius* IBB3154 genome

**Fig 1:**
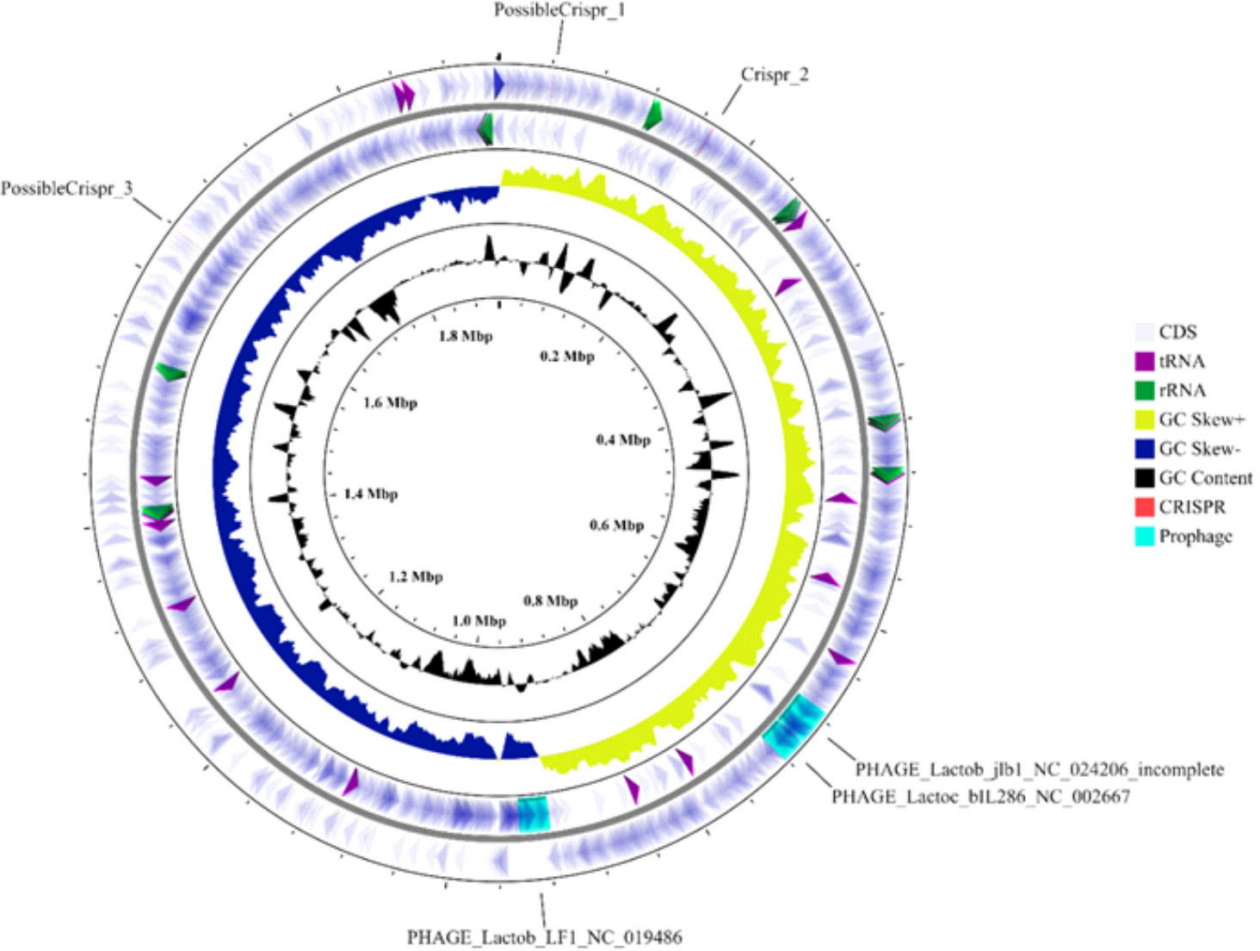
Circular map of the *L. salivarius* IBB3154 chromosome. The two first outer circles indicate predicted genes on the forward and reverse strands. rRNA and tRNA genes are depicted, respectively, by green and purple arrows. The black circle shows GC content, and the yellow/navy blue circle represents GC skew.

To facilitate transcriptomic analysis, we determined the complete genome sequence of *L. salivarius* IBB3154. In addition to the circular chromosome of 1,921,419 bp, two circular plasmids were identified. pIBB3154_1 is a 230,637-bp megaplasmid, while pIBB3154_2 is significantly smaller (13,457 bp). The guanine-cytosine (GC) content of the chromosome is on average 33% and, respectively, 32.3% and 32.2% for pIBB3154_1 and pIBB3154_2 (Fig. 1; Table 1). These values are in agreement with the GC content in *L. salivarius* subsp., which, among other lactobacilli, evolves under the strongest A + T bias (https://www.ncbi.nlm.nih.gov/genome/microbes/).

**TABLE 1:** Features of *L. salivarius* IBB3154 genome

Feature	Value
Chromosome	
Length (bp)	19,214,193
GC content (%)	33
Total genes	1,894
Protein-coding genes	1,794
Genes assigned to COGs	1,248
rRNA genes	22
tRNA genes	75
Prophages (incomplete) CRISPR locus (possible)	2 (1) 1 (2)
Plasmid pIBB3154_1	
Length (bp)	230,637
GC content (%)	32.3
Total genes	220
Protein-coding genes	220
Genes assigned to COGs	112
Plasmid pIBB3154_2	
Length (bp)	13,457
GC content (%)	32.2
Total genes	15
Protein-coding genes	15
Genes assigned to COGs	6

A total of 1,894 genes were found on the chromosome, of which 1,794 were annotated as protein-coding genes. At the origin of replication, there is a clear-cut GC skew and skewed distribution of genes on the two DNA strands, so that their transcription is in the same direction as the replicative fork migration (Fig. 1). Seventy-five functional tRNA genes representing all amino acids and seven complete rRNA operons were identified, four on the forward strand and three on the reverse strand. Six rRNA operons are gathered in the vicinity of some of the tRNA genes (Fig. 1). All rRNA operons and most tRNA genes are organized in the direction of fork migration.

A total of 1,280 genes from the entire chromosome (~70%) had a putative biological function, while the other ~30% genes were assigned as hypothetical or of unknown function. Out of the total of 1,894 predicted proteins, 1,248 (65.9%) could be assigned to COGs functional category. About 11% of these proteins are involved in cellular processes and signaling (categories D, M, N, O, and T), 20% in information storage and processing (categories J, K, and L), 25% in metabolism (categories C, E, F, G, H, I, P, and Q), and 12% are poorly characterized (categories R and S) (Table S4).

As many as 51% of proteins encoded by 116 genes in pIBB3154_1 could not be assigned to any COG functional category; 9% are involved in cellular processes and signaling (categories D, M, O, T, and V), 14% in information storage and processing (J, K, and L), 18% in metabolism (C, E, F, G, I, and P), and 7% are poorly characterized (R and S). Similarly, 60% of the proteins encoded by 15 genes in pIBB3154_2 are also not included in any of the COG categories, while others are involved in cell wall/membrane/envelope biogenesis (COG category M), transcription (K), and replication, recombination, and repair (L) (Table S3). In both plasmids, genes belonging to the L category are the most strongly represented, including those coding for integrases, recombinases, and transposases/insertion elements. The occurrence of such mobile sequences in plasmids is a rather common feature, also in plasmids from *Lactobacillus* spp. (37). and indicate that many of the plasmid-located operons could undergo recombination events within this genus or even beyond.

A widespread presence of prophages in *Lactobacillus* spp. has been reported (38) and IBB3154 was usual in this respect. One incomplete (27.2 kb) and two intact prophage regions ranging in length from 18.6 to 26.6 kb were identified (Fig. 1). One of them contains a phage attachment site and another harbors an *IBB3154_0897* gene that encodes a putative phage lysin, indicating that this phage may undergo the lysogenic pathway.

CRISPR are a sort of bacterial immune system that confers resistance against mobile genetic elements. CRISPRs are often located adjacent to *cas* genes encoding Cas (CRISPR-associated) proteins (39). The Type IIA CRISPR-*cas* system (CRISPR_2) and two possible CRISPRs are present in the IBB3154 genome (Fig. 1). The CRISPR_2 locus contains six conserved direct repeats of a 36-bp-long sequence with the consensus of 5′ GTTTCAGAAGTATGTTAAATCAATAAGGTTAAGACC 3′ preceded by four *cas* genes (*IBB3154_0123*, *IBB3154_0124*, *IBB3154_0125*, and *IBB3154_0126*) encoding, respectively, the Cas9, Cas1, Cas2, and Csn2 proteins.

The CARD database detected only one gene (*vanT*), whose product is involved in antibiotic resistance. No genes associated with virulence or pathogenicity factors were identified in the genome of *L. salivarius* IBB3154 [according to VFDB (40)].

### Transcriptome analysis of *L. salivarius* IBB3154

For comparative transcriptomic analysis, the RNA-seq methodology was used. Total RNA was isolated from bacterial cells growing at 42°C and 37°C. The transcriptome library was constructed, and the resulting amplified cDNA library was sequenced. The RNA-seq reads were aligned to the complete genome sequence of the *L. salivarius* IBB3154 strain, and to identify DEGs, we analyzed the count data from RNA-seq sequencing assays and identified differentially expressed genes (Table 2). Comparative transcriptomic RNA-seq resulted in the identification of genes whose expression was different in bacterial cells growing at 42°C and 37°C. The expression level of the 11 genes was approximately twice as high at 42°C compared to their expression at 37°C. Five genes showed twice as much expression at 37°C. The classification of genes showing different expressions in the studied temperatures by Kyoto Encyclopedia of Genes and Genomes pathway analysis revealed that they are involved in numerous biological pathways, including metabolism—degradation of glycerol (dihydroxyacetone binding subunit, *dhaK*), purine metabolism (adenine deaminase), phosphonate and phosphinate metabolism (choline-phosphate cytidylyltransferase), and osmoregulation (*opuD*). We paid attention to two genes that both had a high level of expression and showed differences in the level of expression depending on the culture temperature used (Table 2). These genes encode the predicted cell wall-anchored proteins SasA1 and SasA2. A comparison of the amino acid sequences of the SasA1 and SasA2 proteins with the sequences available in the databases indicates that they belong to the group of serine-rich repeat proteins (SRRP). These proteins in gram-positive bacteria mediate adherence to various surfaces of the host. Many of them are virulence factors that contribute to bacterial pathogenesis and biofilm formation (41 – 43). SRRP proteins are characterized by highly glycosylated serine-rich regions and one or two species-unique regions (NR domains) located at the N-terminus. At the C-terminus, they contain a hydrophobic region, a positively charged tail, and a sortase family LPXTG motif recognized by surface peptidases, which catalyze the cleavage of the threonine-glycine bond and then covalently bind the protein to the peptidoglycan layer (43). The export of SRRP proteins to the surface of bacterial cells takes place using SecY2A2, after recognition of the signal peptide sequence located at the N-terminus. This secretion system (aSec) consists of the SecA2 protein, the SecY2 translocon channel, and three to five additional Sec proteins (Asp1–5). Genes encoding these proteins are usually found in the same gene cluster as the *srrp* gene (44). Searching the genomic sequence of strain *L. salivarius* IBB3154 showed that in the vicinity of the *sasA1* gene there are genes that build the secretion system as well as genes involved in the O-glycosylation process.

**TABLE 2:** Proteins encoded by temperature-regulated genes of *Ligilactobacillus salivarius* IBB3154^*a*^

Feature ID	EDGE test: 37°C_RPKM vs 42°C_RPKM, tagwise dispersions		Means	
	Fold change	*P*-value	37°C_RPKM	42°C_RPKM
Glycine betaine transporter OpuD	3.48	0	51.16	169.83
Choline-phosphate cytidylyltransferase (EC 2.7.7.15)	3.46	0.0001	84.02	270.69
Adenine deaminase (EC 3.5.4.2)	3.29	0.0538	206.11	561.18
Phosphoenolpyruvate-dihydroxyacetone phosphotransferase (EC 2.7.1.121), dihydroxyacetone binding subunit DhaK	2.28	0.0052	153.07	332.66
**Predicted cell-wall-anchored protein SasA (LPXTG motif)_1**	**2.15**	**0.0004**	**623.59**	**1,231.86**
Pyruvate dehydrogenase E1 component alpha subunit (EC 1.2.4.1)	1.97	0.0057	296.67	540.03
Amino acid permease AapA	1.91	0.0098	196.61	349.20
Phosphoenolpyruvate-dihydroxyacetone phosphotransferase (EC 2.7.1.121), ADP-binding subunit DhaL	1.90	0.0239	67.29	121.68
**Predicted cell-wall-anchored protein SasA (LPXTG motif)_2**	**1.88**	**0.0052**	**2,761.57**	**4,612.54**
PTS system, mannose-specific IIA component/PTS system, mannose-specific IIB component (EC 2.7.1.69)_1	1.82	0.0154	506.62	870.87
PTS system, mannitol-specific IIC component/PTS system, mannitol-specific IIB component (EC 2.7.1.69)	1.81	0.039	134.95	230.57
Transcriptional regulator, MarR family_3	−1.90	0.0044	187.10	92.11
Stress-responsive transcriptional regulator PspC	−1.97	0.0402	112.33	55.65
Transcriptional regulator, MerR family_1	−2.04	0.0099	55.16	25.65
Hypothetical protein_58	−2.19	0.0219	61.60	26.19
Aggregation-promoting factor_2	−2.32	0.0114	376.18	148.77

^*a*^ Transcriptomic analysis of a culture grown at 37°C and at an elevated temperature of 42°C. Boldface type indicates chosen promoters. As the criteria for a significant gene expression difference, the threshold was used at a fold change of 1.8 and a *P*-value < 0.05.

In addition, we identified genes that are highly expressed in cultures grown at both temperatures. Proteins encoded by those genes (Table 3), mainly involved in basic metabolic pathways or essential for the maintenance of basic cellular functions, are highly conserved. What is interesting is that some of them are also known as moonlighting proteins, which means that besides their canonical functions, they were also adopted for other activities. GAPDH (glyceraldehyde 3-phosphate dehydrogenase) is a ubiquitous enzyme involved in glycolysis. As a surface-associated protein, GAPDH in many pathogenic bacteria can bind to host proteins such as fibronectin, plasminogen, or laminin. The disruption of the extracellular matrix facilitates bacterial dissemination within the host organism (45). Conversely, when located extracellularly, GAPDH exerts immunomodulatory effects, influencing both innate and acquired immunity (46, 47). Similarly, FBA (fructose-bisphosphate aldolase), an enzyme of the glycolytic pathway, promotes adhesion to host cells (48). Despite its highly conserved function in protein synthesis, non-canonical functions have also been described for elongation factor Tu (Ef-Tu). The cell surface-associated Ef-Tu mediates the attachment of *Lactobacillus johnsonii* NCC533 (La1) to human intestinal cells and mucins (49). Enolase, generally known as the cytoplasmic enzyme involved in glycolysis and gluconeogenesis, was found in *Lactobacillus gasseri* ATCC 33323 associated with the cell surface, which inhibits the adhesion of the sexually transmitted pathogen, *Neisseria gonorrhoeae*, to epithelial cells (50).

**TABLE 3:** Proteins encoded by highly expressed genes of *Ligilactobacillus salivarius* IBB3154 in both temperatures^*a*^

Feature ID	EDGE test: 37°C_RPKM vs 42°C_RPKM, tagwise dispersions		Means	
	Fold change	*P*-value	37°C_RPKM	42°C_RPKM
FIG00747762: hypothetical protein	1.24	0.41	45,041.16	47,960.24
Translation Ef-Tu	1.15	0.52	39,775.23	40,658.45
NAD-dependent glyceraldehyde-3-phosphate dehydrogenase (EC 1.2.1.12)	1.34	0.16	27,828.06	33,320.78
Fructose-bisphosphate aldolase class II (EC4.1.2.13)	1.21	0.44	19,177.49	20,131.37
Enolase (EC 4.2.1.11)	1.33	0.21	13,312.54	15,615.92

^*a*^ Transcriptomic analysis of culture grown at 37°C and 42°C.

### Strength analysis of selected promoters using a reporter gene at 37°C and 42°C

**Fig 2:**
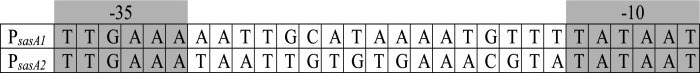
Predicted promoter sequences for the *sasA1* and *sasA2* genes. The −10 and −35 regions, spaced 16 nucleotides apart, are identical to the analogous nucleotide sequences of the consensus regions of the gram-positive bacterial promoters (−10: TATAAT, −35: TTGACA) (according to PePPER, University of Groningen, The Netherlands).

As the level of gene expression depends, among other things, on the efficiency of promoter, we decided to analyze the upstream region of genes encoding the above-mentioned proteins. To analyze the strength of the promoters, we used the *gus* reporter gene, which encodes β-glucuronidase. For the β-glucuronidase activity test, we chose promoters of genes encoding predicted cell wall-anchored protein SasA1 (P*_sas1_*) and predicted cell wall-anchored protein SasA2 (P*_sas2_*), which were shown in the comparative transcriptomic assay as temperature inducible. Based on the analysis of the nucleotide sequence preceding the *sasA1* and *sasA2* genes, the potential promoter sequences were determined: −10 and −35, which is largely consistent with the consensus established for *Lactobacillus* sp. genes read by RNA polymerase with the main sigma factor (σ70) (Fig. 2). Additionally, we analyzed the promoter of the gene encoding fructose-bisphosphate aldolase (P*_fab_*), as the transcriptome analyses showed a high level of its expression. Attempts to clone the estimated promoter of high-expression genes: hypothetical protein FIG00747762 (P*_hp762_*), NAD-dependent glyceraldehyde-3-phosphate dehydrogenase (P*_NAD_*), and enolase (P*_eno_*), into pNZ8008 have failed. We assume that the promoters were too strong, and therefore the high expression of the reporter gene was toxic for *Ligilactobacillus* sp. cells.

**Fig 3:**
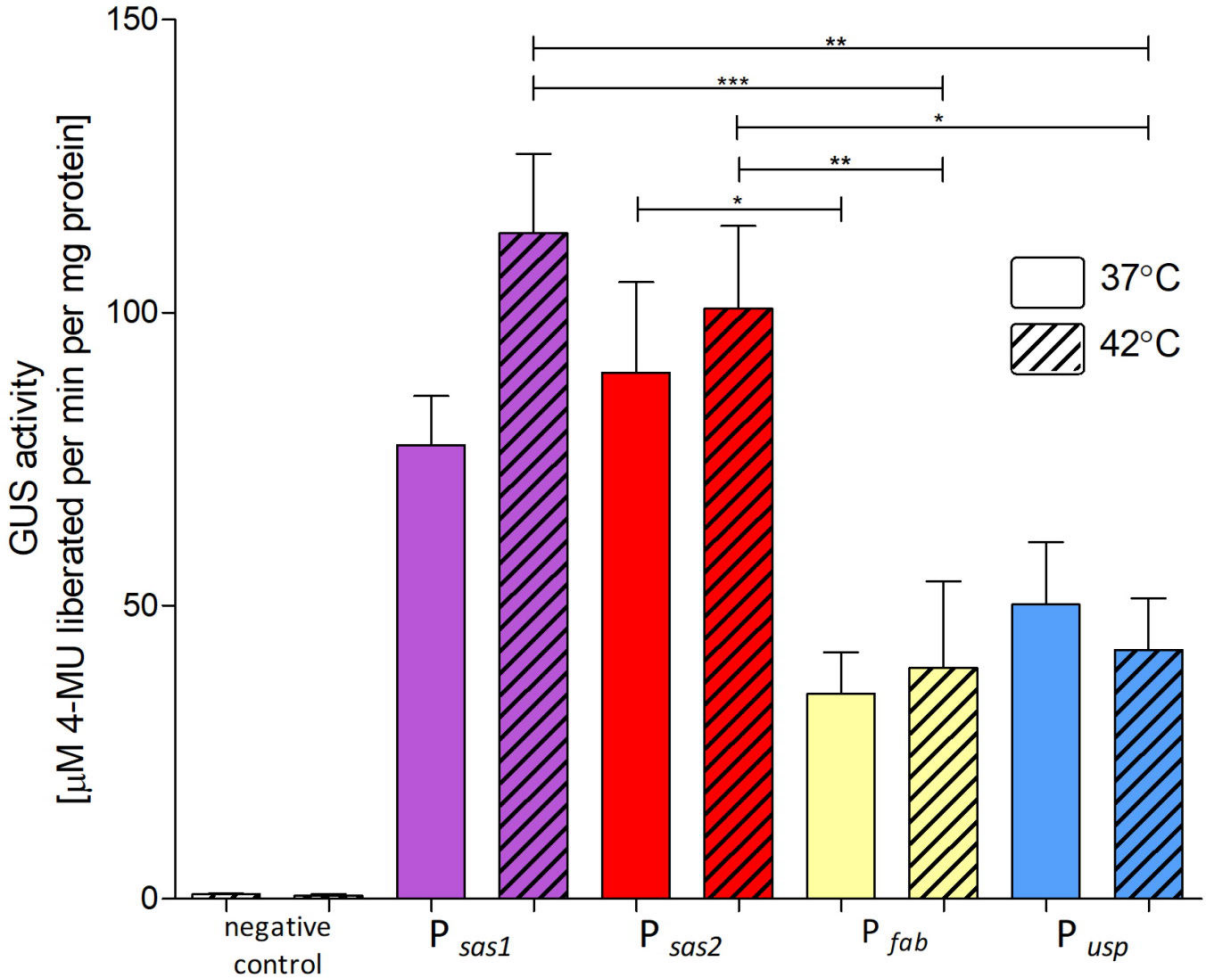
The specific activity of β-glucuronidase in the *L. salivarius* IBB3154 cells carrying the constructed vectors. The presented results are mean values from three independent trials. Differences between 37°C and 42°C were not statistically significant. Differences that are statistically significant are marked by an asterisk (*P* < 0.05).

As the base for constructing a series of expression vectors, we used the derivative of the pSH71 plasmid, pNZ8008, in which the β-glucuronidase gene was cloned under the control of the nisin-induced promoter, P*_nis_*. It was confirmed that the pSH71 replicon can replicate in *L. salivarius* IBB3154 cells. In constructed plasmids, we replaced P*_nis_* with the analyzed promoters P*_sas1_* (pUWM1491), P*_sas2_* (pUWM1481), and P*_fab_* (pUWM1498). β-glucuronidase activity was tested in *L. salivarius* IBB3154 cells harboring the expression vectors grown at 42°C and 37°C. As a negative control, we used *L. salivarius* carrying pNZ8008 cultured in a medium without the addition of nisin. As a positive control, we used cells of the *L. salivarius* IBB3154 strain harboring the vector-coding β-glucuronidase under the control of a commonly used promoter of the *usp45* gene (pUWM1565), described as a strong promoter derived from strains of *Lactoccocus lactis* (Fig. 3) (23, 51).

In addition, there was no difference in the β-glucuronidase expression between the two promoters even though the transcriptomics indicated that the *sasA2* promoter was approximately four times stronger than the *sasA1* promoter. However, the data presented indicate that both promoters—P*_sasA1_* and P*_sasA2_* —are strong promoters with high activity at both temperatures. The promoter of the gene encoding fructose-bisphosphate aldolase (P*_fab_*) was characterized by much lower activity.

### Strength analysis of selected promoters using a reporter gene in the presence of bile salts

In the digestive tract, lactic acid bacteria encounter various stressful conditions. Intraluminal pH conditions change from slightly acidic in the crop (4.5–6.5), through highly acidic in the proventriculus and gizzard (1.9–4.5), to a level above six in the intestine (52). Additionally, the gastrointestinal tract (GI) has an osmolarity equivalent to 0.3 mol/L of NaCl (53). Moreover, LABs are exposed to bile acids, compounds essential for digestion and nutrient absorption. Determining whether selected promoters retain their activity in the intestinal environment is crucial for creating a vaccine-antigen-producing strain.

**Fig 4:**
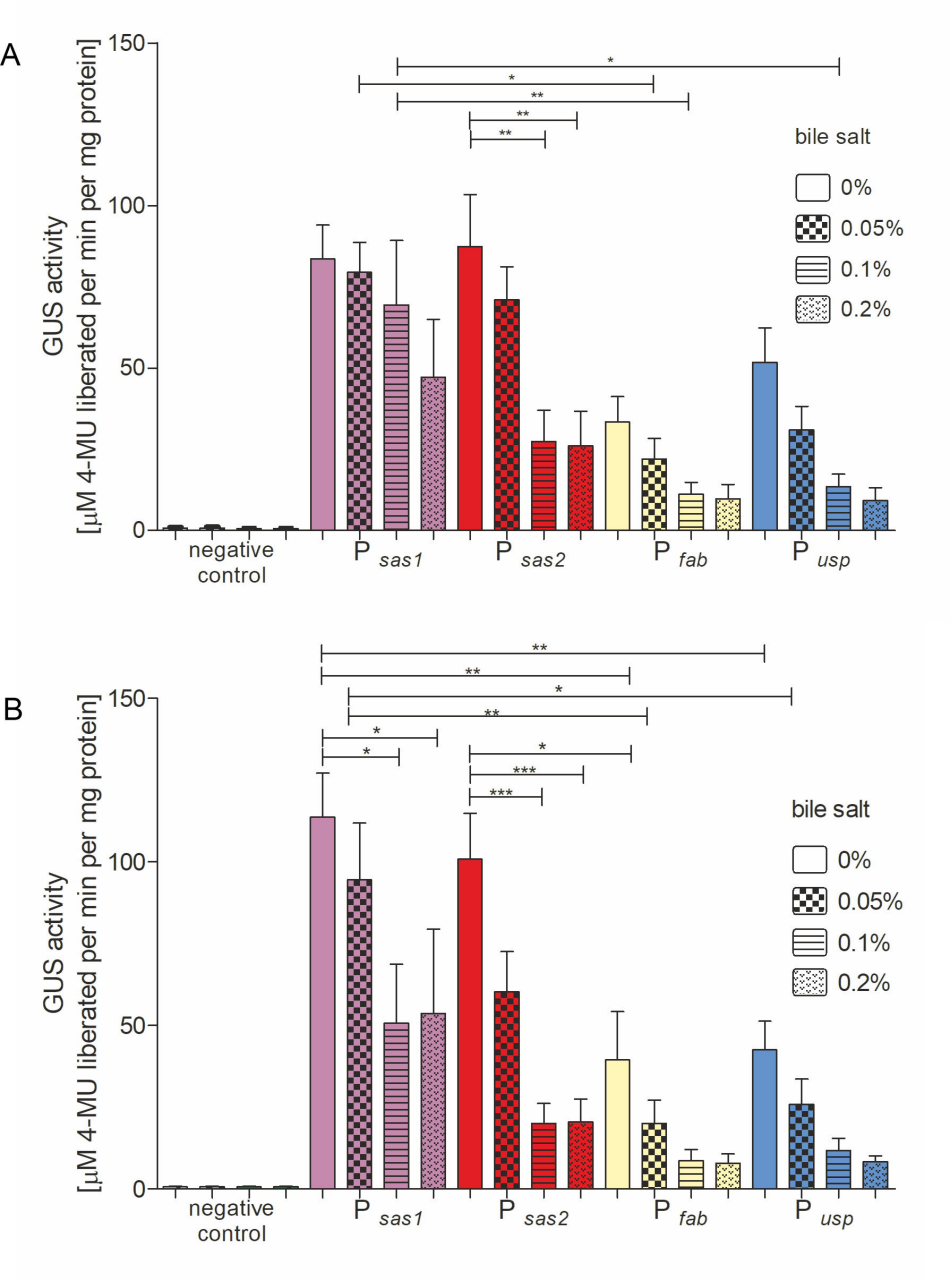
The specific activity of β-glucuronidase in the *L. salivarius* IBB3154 cells carrying the constructed vectors in the presence of various concentrations of bile salts: (A) 37°C and (B) 42°C. The presented results are mean values from three independent trials. Differences that are statistically significant are marked by an asterisk (*P* < 0.05).

The activity of the reporter gene was assessed in the presence of bile salts. Addition of bile salts reduces the strength of the tested promoters. However, in the *Ligilactobacillus* sp. strains that carry the plasmids pUWM1498 (P*_fab_*) and pUWM1565 (P*_usp45_*), the expression of the reporter gene is inhibited at a concentration of 0.1%. In the case of the *Ligilactobacillus* strain that carries the plasmid containing the promoter sequence of the *sas1* gene, β-glucuronidase activity reaches the level of P*_fab_* activity observed in medium without the addition of bile salts, and the activity level is low (Fig. 4).

## DISCUSSION

Representatives of the *Ligilactobacillus* genus exhibit significant physiological and genetic diversity. The selection of an appropriate strain for use as an antigen carrier is a task that requires special care. Strains should not harbor any acquired antimicrobial resistance genes to clinically relevant antimicrobial compounds. The presence of genes conferring antibiotic resistance in the genome carries the risk of spreading resistance within the microbiome of animals' gastrointestinal tracts and in the environment in general. An example of the transfer of tetracycline resistance genes from *L. plantarum* and *Lactobacillus delbruekii* subsp. Bulgaricus to the pathogenic *Listeria monocytogenes* has been documented by Yang and Yu (54). *L. salivarius* IBB3154 strain has the ability to effectively colonize the gastrointestinal tract of chickens, making it a potential candidate for use as a live bacterial vector in these animals (24). In the genome of the IBB3154 strain, only a gene conferring resistance to vancomycin was identified (according to the CARD database). It is worth noting that vancomycin resistance is characteristic of many strains of lactic acid bacteria. This resistance is intrinsic, chromosomally encoded, and not inducible or transferable.

In the construction of an effective vaccine, the amount of antigen produced may play a key role. For protein expression in LAB, constitutive promoters, e.g., P*_ldhl_* (lactate dehydrogenase promoter), P*_slpA_* (promoter of the gene encoding the protein SlpA), and P_tu_ (Ef-Tu promoter) (55 – 58), are often used. Induced promoters provide control over gene expression, which is important when the gene product is toxic to the host. One of the best-known examples is the promoter used in the Nisin-Controlled Gene Expression System, induced by the addition of the anti-bacterial peptide nisin to the medium. However, its drawback is the inability to use nisin *in vivo,* which results in antigen production occuring only during the strain preparation (59). Carbohydrate-induced promoters have also been identified (P*_fos_*, P*_tre_*). Attention is drawn to the promoter activated by the presence of fructoligosaccharide (P*_fos_*), a prebiotic that stimulates the development of intestinal microbiota (60). An alternative may be promoters induced by stress conditions prevailing in the host’s digestive tract (1, 61, 62). A very good example is the P*_groESL_* promoter, which was used to develop a new inductive gene expression system called SICE (Stress-Inducible Expression System). The same research group developed an *in vivo* LIVE-induced system (*Lactobacilli In Vivo* Expression System), which was used to express anti-inflammatory interleukin 10 (IL-10) and glucan-binding protein B *Streptococcus mutans* (GbpB) (63).

The presented study was intended to identify promoters that could be used to control the expression of heterologous proteins in *Ligilactobacillus* cells. Therefore, the strength of promoters, selected on the basis of a comparison of the transcriptomes of the *L. salivarius* IBB3154 strain grown at 37°C and 42°C, was investigated. For those analyses, we selected P*_sasA1_*, P*_sasA2_*, and P*_fab_* promoters, as they showed higher potency at 42°C, which corresponds to the body temperature of the birds. Additionally, we analyzed the P*fab* promoter, as the transcriptome analyses indicated a high level of its expression, both at 37°C and 42°C. To evaluate whether the screened promoters can be used to enhance transcription of genes encoding antigens, the three promoters were separately integrated upstream of the reporter gene *gusA*. As the base module for the creation of constructs, we used the pNZ8008 plasmid, which contains the broad-host range replicon and harbors the strong and nisin-inducible P*_nis_* promoter controlling the expression of β-glucuronidase. The P*_usp45_* promoter was used as a positive control (64). The tests were carried out at two temperature variants: 37°C and 42°C. The presented results show that the P*_sasA1_* and P*_sasA2_* promoters are characterized by the highest activity under the tested conditions. At 37°C, an enhanced strength of the promoter of the gene encoding the SasA1 protein is observed, while at 42°C, the P*_sasA2_* promoter seems to be more active. We assume that both promoters (P*_sasA1_* and P*_sasA2_*) are strong enough to place genes encoding hybrid proteins under their control, but their strength is not affected by temperature.

In the digestive tract, lactic acid bacteria are exposed to acids and bile salts. Bile salt concentrations in the small intestine fluctuate between 0.2 and 2%, resulting in lower concentrations in the large intestine (65). Some LABs have the ability to survive under these conditions and to colonize the gut, which is important in the production of orally administered vaccines. The activity of promoters in the presence of bile salts is crucial for the production of heterologous proteins by lactic acid bacteria. What is more, as these genes encode proteins most likely arising at the adhesion site, the strength of their promoters in the presence of bile salts was analyzed. Bile salts at concentrations of 0.05%, 0.1%, and 0.2% were used in the research. The results show that bile salts and their concentration have an impact on the strength of the tested promoters. The decrease in the activity of these promoters is already observed at 0.05% salt concentration, while at 0.1% concentration, inhibition of the *gusA* reporter gene expression is observed in the case of P*_fab_* and P*_usp45_* promoters. P*_sasA1_* and P*_sasA2_* promoters were most potent among the studied genes, as in the experiment with temperature variants; however, bile salts did not induce the increased activity of the tested promoters. It is worth noticing that Martínez-Fernández et al. (66) identified bile salt-induced promoters in *Lactobacillus casei* BL23 and *Lactobacillus plantarum* WCFS1. The authors constructed the pNZ vector:16090-aFP, which could be used as a gene carrier and be utilized in the construction of vaccines administered to the mucous membranes. The same salt concentrations were selected for the tests as in the presented work, i.e., 0.05%, 0.1%, and 0.2%. Induction of the *L. casei* BL23-derived P16090 promoter was observed in the starting strain *L. casei* BL23 as well as in *L. plantarum* WCFSI, *L. rhamnosus,* and *L. reuteri*, i.e., in the strains most often used in the probiotic industry (66).

A similar approach was applied in the cases of *L. salivarius* and *Ligilactobacillus agilis*. In the study conducted by Wang et al. (67), the primary objective was to identify genes whose expression changes in response to the presence of bile salts. Among the genes whose expression increased were those encoding ABC transporters, genes encoding enzymes involved in surface charge modification, and enzymes necessary for maintaining redox homeostasis. These studies serve as an excellent starting point for both elucidating the molecular mechanisms associated with the response to bile stress and searching for a strong promoter active in the gastrointestinal tract (67). Vezina et al. (68) also employed transcriptomics to identify strong constitutive promoters in *L. agilis* cells. The most abundant transcripts originated from genes encoding glyceraldehyde-3-phosphate dehydrogenase (GAPDH), translational Ef-Tu, enolase, a putatively secreted protein, and a cell wall-associated hydrolase (68). It is worth noting that three of these genes were also identified in our own research.

Techniques such as RNA-seq, DNA microarrays, or proteomics allow the development of inducible, repressive, or constitutive expression vectors, but in this case, the analysis using the reporter’s gene did not confirm the results obtained from the comparison of the transcriptome of the *L. salivarius* strain at 37°C and 42°C. The P*_sasA2_* promoter exhibits diminished activity at elevated bile salt concentrations (0.1% and 0.2%) at both 37°C and 42°C, while the activity of P*_sasA1_*, although slightly lower at 42°C, remained at a relatively high level. These results suggest the potential utility of the *sasA1* gene promoter for the expression of *Campylobacter* genes encoding immunogenic proteins.

## Data Availability

The whole genome project and RNA-seq data have been deposited at GenBank under BioProject number PRJNA434808 (accession numbers CP027644 to CP027646, SRX20850324 to SRX20850329).

## SUPPLEMENTAL MATERIAL

Table S1 spectrum.02844-23-s0001.docx Bacterial strains and plasmids.

Table S2 spectrum.02844-23-s0002.docx Oligonucleotides.

Table S3 spectrum.02844-23-s0003.docx Statistics of RNA-seq samples.

Table S4 spectrum.02844-23-s0004.docx COG functional categories.

ASM does not own the copyrights to Supplemental Material that may be linked to, or accessed through, an article. The authors have granted ASM a non-exclusive, world-wide license to publish the Supplemental Material files. Please contact the corresponding author directly for reuse.
