# Supplementary material for: Genomic and transcriptomic analysis of Ligilactobacillus salivarius IBB3154—in search of new promoters for vaccine construction
Source: Microbiol Spectr. 2023 Nov 20;11(6):e02844-23. doi: 10.1128/spectrum.02844-23 (PMC10715006; doi:10.1128/spectrum.02844-23)
Supplement: Table S1 — Bacterial strains and plasmids. [file spectrum.02844-23-s0001.docx]

**Table S1.** Bacterial strains and plasmids used in this study.

| **Strain or plasmid** | **Relevant phenotype(s) or genotype(s)** | **Source or reference(s)** |
| --- | --- | --- |
| **Strains** | | |
| *L. salivarius* IBB3154 | Isolated from hen feaces | (69) |
| *E. coli* TG1 | *supE thi1* Δ(*lac^-^proAB*) Δ(*mcrB-hsdSM*)*5* (*rK^-^ mK^-^*) F' [*traD36 proAB^+^ lacI^q^ lacZ*Δ*M15*] | (29) |
| *E. coli* MC1061 | *araD139*, Δ(*ara, leu*)7697, Δ*lacX74*,g*alU*^-^, *galK*^-^, *hsr*^-^, *hsm*^+^, *strA* | (70) |
| *L. lactis* subsp. *lactis* IL1403 | Laboratory strain | (71) |
| **Plasmids** | | |
| pJET1.2 blunt | AMP^R^; vector for cloning PCR products | Thermo Fisher Scientific |
| pBluescript II SK+ | AMP^R^; general cloning vector | Stratagene |
| pNZ8008 | CHL^R^, pSH71 rolling circle replicon (broad host range); vector for β-glucuronidase activity measurement | MoBiTec  (72) |
| pUWM1476 | Promoter of *sas1* gene (P*_sas1_*) in pBluescript II SK+ | This study |
| pUWM1477 | Promoter of *sas2* gene (P*_sas2_*) in pBluescript II SK+ | This study |
| pUWM1481 | Gene encoding β-glucuronidase under P*_sas2_* promoter in pNZ8008 | This study |
| pUWM1486 | fragment containing promoter of *sas1* gene (P*_sas1_*) in pJET 1.2 | This study |
| pUWM1491 | Gene encoding β-glucuronidase under P*_sas1_* in pNZ8008 | This study |
| pUWM1488 | fragment containing promoter of fructose-bisphosphate aldolase (P*_fbaA_*) in pJET 1.2 | This study |
| pUWM1498 | Gene encoding β-glucuronidase under P*_fbaA_* in pNZ8008 | This study |
| pUWM1553 | Fragment containing P*_usp45_* in pJet 1.2 blunt | This study |
| pUWM1565 | Gene encoding β-glucuronidase under P*_usp45_* in pNZ8008 | This study |

References

29. Hanahan D. 1983. Studies on transformation of *Escherichia coli* with plasmids. J Mol Biol 166:557–580. https://doi.org/10.1016/s0022-2836(83)80284-8

69. Kobierecka P, Wyszyńska A, Maruszewska M, Wojtania A, Żylińska J, Bardowski J, Jagusztyn-Krynicka EK. 2015. Lactic acid bacteria as a surface display platform for *Campylobacter jejuni* antigens. Microb Physiol 25:1–10. https://doi.org/10.1159/000368780

70. Casadaban MJ, Cohen SN. 1980. Analysis of gene control signals by DNA fusion and cloning in *Escherichia coli*. J Mol Biol 138:179–207. https://doi.org/10.1016/0022-2836(80)90283-1

71. Bolotin A, Wincker P, Mauger S, Jaillon O, Malarme K, Weissenbach J, Ehrlich SD, Sorokin A. 2001. The complete genome sequence of the lactic acid bacterium *Lactococcus lactis* ssp. *lactis* IL1403. Genome Res 11:731–753. https://doi.org/10.1101/gr.gr-1697r

72. de Ruyter PG, Kuipers OP, de Vos WM. 1996. Controlled gene expression systems for *Lactococcus lactis* with the food-grade inducer nisin. Appl Environ Microbiol 62:3662–3667. https://doi.org/10.1128/aem.62.10.3662-3667.1996
