# Supplementary material for: Genomic and transcriptomic analysis of Ligilactobacillus salivarius IBB3154—in search of new promoters for vaccine construction
Source: Microbiol Spectr. 2023 Nov 20;11(6):e02844-23. doi: 10.1128/spectrum.02844-23 (PMC10715006; doi:10.1128/spectrum.02844-23)
Supplement: Table S2 — Oligonucleotides. [file spectrum.02844-23-s0002.docx]

**Table S2.** Oligonucleotides used in this study.

| **Name of primer** | **Sequence 5’→3’*** | **Restriction recognition site** |
| --- | --- | --- |
| pUWM1476_BglII | **TAGAACTAGTGGATCCCCCGGGC** | - |
| pUWM1476_PstI | TCCTGCAG**ATGAATTCTGAGCTCTTAAATAC** | PstI |
| Usp45_BglII | AGAAGATCT**TGTTTACCAGCTAGCGCCTA** | BglII |
| Usp45_PstI | AGCCTGCAG**GAGAGTACCACTGTAGACAT** | PstI |
| Faldo_BglII | AGATCTTTGCTAAGGCAGTAAGAAATATC | BglII |
| Faldo_PstI | TCTGCAGATTACATCTTAATTACTCTTCTG | PstI |

*∗*Bold letters indicate *Lactococcus*/*Ligilactobacillus* nucleotide sequences, and the restriction recognition sites introduced for cloning purposes are underlined. Primers were based on the *Ligilactobacillus salivarius* IBB3154 and *Lactococcus lactis* IL1403 nucleotide sequences.
