# Supplementary material for: Genomic and transcriptomic analysis of Ligilactobacillus salivarius IBB3154—in search of new promoters for vaccine construction
Source: Microbiol Spectr. 2023 Nov 20;11(6):e02844-23. doi: 10.1128/spectrum.02844-23 (PMC10715006; doi:10.1128/spectrum.02844-23)
Supplement: Table S3 — Statistics of RNA-seq samples. [file spectrum.02844-23-s0003.docx]

**Table S3.** Information regarding read counts of RNASeq samples.

| **Sample name** | **Accession** | **Total number of raw read counts** | **Number of reads mapped against *L.salivarius* 3154 (accession: CP027644)** | **Percentage of**  **mapped reads** |
| --- | --- | --- | --- | --- |
| 3154-37-1 | SRR25097015 | 6,632,958 | 5,340,543 | 80,79 |
| 3154-37-2 | SRR25097014 | 4,529,285 | 4,034,350 | 89,23 |
| 3154-37-3 | SRR25097013 | 12,549,372 | 10,207,557 | 81,69 |
| 3154-42-1 | SRR25097012 | 4,536,672 | 4,271,079 | 94,37 |
| 3154-42-2 | SRR25097011 | 7,203,211 | 6,582,756 | 91,64 |
| 3154-42-3 | SRR25097010 | 7,572,776 | 6,732,889 | 90,18 |
