# Supplementary material for: Genomic and transcriptomic analysis of Ligilactobacillus salivarius IBB3154—in search of new promoters for vaccine construction
Source: Microbiol Spectr. 2023 Nov 20;11(6):e02844-23. doi: 10.1128/spectrum.02844-23 (PMC10715006; doi:10.1128/spectrum.02844-23)
Supplement: Table S4 — COG functional categories. [file spectrum.02844-23-s0004.docx]

| **Table S4.** Number of *L. salivarius* IBB3154 genes assigned to general COG functional categories. | | | | | | |
| --- | --- | --- | --- | --- | --- | --- |
| **COG functional class** | **Chromosome** | | **Plasmid pIBB3154_1** | | **Plasmid pIBB3154_2** | |
|  | **№ ORFs** | **% ORFs** | **№ ORFs** | **% ORFs** | **№ ORFs** | **% ORFs** |
| **Metabolism** | **444** | **24.76** | **45** | **18.02** | **0** | **0** |
| C - Energy production and conversion  E - Amino acid transport and metabolism  F - Nucleotide transport and metabolism  G - Carbohydrate transport and metabolism  H - Coenzyme transport and metabolism  I - Lipid transport and metabolism  P - Inorganic ion transport and metabolism  Q - Secondary metabolites biosynthesis, transport and catabolism | 55  104  64  95  31  40  49  6 | 3.07  5.80  3.57  5.30  1.73  2.23  2.73  0.33 | 8  14  1  16  -  1  5  - | 3.51  6.14  0.44  5.30  -  0.44  2.19  - | -  -  -  -  -  -  -  - | -  -  -  -  -  -  -  - |
| **Cellular processes and signalling** | **232** | **12.93** | **20** | **8.67** | **1** | **6.67** |
| D - Cell division and chromosome partitioning  M - Cell envelope biogenesis, outer membrane  N - Cell motility  O - Posttranslational modification, protein turnover, chaperones  T - Signal transduction mechanisms  U - Intracellular trafficking, secretion, and vesicular transport  V - Defence mechanisms | 21  99  4  44  30  12  22 | 1.17  5.52  0.22  2.45  1.67  0.67  1.23 | 1  2  -  7  4  -  6 | 0.44  0.88  -  3.07  1.75  -  2.53 | -  1  -  -  -  -  - | -  6.67  -  -  -  -  - |
| **Information storage and processing** | **354** | **19.74** | **31** | **13.6** | **5** | **33.34** |
| B - Chromatin structure and dynamics  J - Translation, ribosomal structure and biogenesis  K - Transcription  L - DNA replication, recombination and repair | 1  134  94  125 | 0.06  7.47  5.24  6.97 | -  2  6  23 | -  0.88  2.63  10.09 | -  -  1  4 | -  -  6.67  26.67 |
| **Poorly characterized** | **218** | **12.15** | **16** | **7.02** | **0** | **0** |
| R - General function prediction only  S - COG of unknown function | 138  80 | 7.69  4.46 | 9  7 | 3.95  3.07 | -  - | -  - |
| **Not in COGs** | **546** | **30.43** | **116** | **50.88** | **9** | **60** |
